# Soluble P-selectin as an inflammatory mediator potentially influencing endothelial activation in people living with HIV in sub-rural areas of Limpopo, South Africa

**DOI:** 10.1371/journal.pone.0310056

**Published:** 2024-11-27

**Authors:** Haskly Mokoena, Sihle E. Mabhida, Joel Choshi, Machoene D. Sekgala, Bongani B. Nkambule, Duduzile Ndwandwe, Zandile J. Mchiza, André P. Kengne, Phiwayinkosi V. Dludla, Sidney Hanser

**Affiliations:** 1 Department of Physiology and Environmental Health, University of Limpopo, Sovenga, South Africa; 2 Non-Communicable Diseases Research Unit, South African Medical Research Council, Tygerberg, South Africa; 3 School of Laboratory Medicine and Medical Sciences, University of KwaZulu-Natal, Durban, South Africa; 4 Cochrane South Africa, South African Medical Research Council, Tygerberg, South Africa; 5 School of Public Health, University of the Western Cape, Bellville, South Africa; 6 Department of Medicine, University of Cape Town, Cape Town, South Africa; 7 Department of Biochemistry and Microbiology, University of Zululand, KwaDlangezwa, South Africa; Icahn School of Medicine at Mount Sinai Department of Pharmacological Sciences, UNITED STATES OF AMERICA

## Abstract

**Objectives:**

There is a growing need to understand the potential role of soluble platelet selectin (sP-selectin) in sustained endothelial activation through increased levels of soluble intercellular adhesion molecule-1 (sICAM-1) and soluble vascular adhesion-1 (sVCAM-1) in people living with HIV (PLWH) on highly active antiretroviral therapy (HAART).

**Methodology:**

This was a cross-sectional study involving PLWH on HAART (n = 55), in comparison to PLWH not on treatment (HAART-naïve) (n = 29), and (iii) HIV negative controls (n = 48) from the Mankweng area in the Limpopo province, South Africa. We quantified serum levels of sP-selectin, together with sICAM-1 and sVCAM-1. Most of the HAART-exposed group were on treatment for <5 years. We further performed frequency distribution and descriptive statistics for categorical variables.

**Results:**

Soluble P-selectin was positively correlated with sVCAM-1 (r = 0.469; p<0.001) in PLWH on HAART, even after adjusting for confounding factor such as age, BMI, and total cholesterol (r = 0.467; p<0.001). Moreover, in PLWH on HAART sP-selecting was independently associated with the release of sVCAM-1 (β = 0.445; p<0.001), even after adjusting for confounders (β = 0.475; p = 0.001). Serum levels of low-density lipoprotein cholesterol (LDL-C) (p = 0.004) and total cholesterol (p<0.001) were significantly higher in PLWH on HAART as compared to the HAART-naïve group.

**Conclusion:**

There is a need for more studies to investigate the role of sP-selectin in promoting endothelial activation and CVD-risk in PLWH on HAART, especially within the sub-Saharan Africa region.

## Introduction

Cardiovascular diseases (CVDs) are the leading global cause of mortality [1]. Annually, approximately 17.3 million lives are lost to CVD-related mortalities worldwide, surpassing mortalities attributed to the human immunodeficiency virus (HIV) [2]. Sub-Saharan Africa remains the epicentre of the human immunodeficiency virus (HIV) pandemic [3], with South Africa alone reporting approximately 7.6 million people living with HIV (PLWH) [4]. Understanding the association between HIV and elevated risk of CVD-related morbidities is gaining momentum [5–7]. However, it is well-established that PLWH consistently present with chronic inflammation, potentially leading to endothelial activation that may predispose them to increased CVD-risk [8]. Nevertheless, highly active antiretroviral therapy (HAART) has proven effective in suppressing the virus in PLWH [9–11]. Despite its effectiveness in viral suppression, more research is needed to understand its potential role in protecting against CVD-related complications [12,13]. Some studies have also suggested that HAART can increase circulating lipid parameters such as triglycerides and low-density lipoprotein cholesterol (LDL-C), which are considered risk factors for the development of CVDs [14–16].

Current evidence highlights the critical role of inflammation in the development of CVDs among PLWH, including those on HAART [17,18]. In a pro-inflammatory state, endothelial activation is accompanied by the release of soluble platelet selectin (sP-selectin) that promotes leukocyte adhesion and initiate damage to the vascular endothelium [11]. This pathophysiological consequence compromises the integrity and permeability of the endothelium, potentially contributing to the initiation of endothelial dysfunction [19–21]. Indeed, sustained endothelial activation is characterized by an increase in soluble intercellular and vascular adhesion molecules-1 (sICAM-1 and sVCAM-1) and surface expression of sP-selectin preceding endothelial dysfunction [22]. Elevated circulating levels of sICAM-1 and sVCAM-1 are consistent with an undesirable inflammatory microenvironment in PLWH [23]. The initiation of HAART may decrease the expression of inflammatory mediators and promote endothelial recovery [12], however long-term studies to prove this are lacking. Emerging evidence suggests that administering HAART in PLWH for approximately six months can suppress inflammation and promote endothelial recovery comparable to individuals without the condition [24]. However, some findings indicate that the beneficial effects of HAART may diminish after six months, leading to increased inflammation and initiation of endothelial dysfunction [12,24,25].

There is a scarcity of literature addressing the potential role of inflammation in contributing to increased CVD-risk through the detrimental effects of endothelial activation in PLWH, especially within sub-Saharan African regions. The primary focus of this study is to investigate the potential role of sP-selectin as an inflammatory mediator driving endothelial activation in PLWH on HAART from the Limpopo province, South Africa. Additionally, the study explores the impact of HAART exposure duration on endothelial function. This holds significance for PLWH confronting a growing burden of CVDs [26], providing a basis to potentially develop interventions that could extend the lives of these individuals.

## Methods and materials

### Study design and setting

This was a cross-sectional study conducted at the Mankweng hospital and referral clinics between February 2018 and December 2020 in the Capricorn District of the Polokwane local municipality in the Limpopo province, South Africa. Participants were outsourced from public clinic which specializes in health promotion through the early detection of diseases, treatment, and prevention, expanding from the previously published study by Hanser et al., (2022) [16]. These clinics incorporated Dikgale, Evelyn Lekganyane, Makanye, Molepo, Mamotswa, Mamabolo, Mothiba, Nobody, and Sebayeng, which were selected for having a large proportion of PLWH on HAART and HIV testing.

### Study population

The study population was made up of adults (≥18 years of age) classified into three groups, namely: (i) PLWH on HAART (n = 55), (ii) PLWH not on treatment (HAART-naïve) (n = 29), and (iii) HIV negative controls (n = 48). It is worth mention that participants were recruited to partake in the study regardless of their HART regimen combination and exposure duration. All participants were selected using a non-probability (subjective) sampling method based on their HIV and treatment status to achieve a population size of 132 participants. The sample size was determined using mathematical equations with reference to a study by Kim et al. (2021) [27] which indicated that the estimated proportion of the population living with HIV in the Limpopo province was 11.18% with a confidence interval (CI) of 95% and a 5% margin of error. The study excluded candidates with existing traditional risk factors of CVDs incorporating hypertension, dyslipidaemia, obesity, diabetes mellitus, coagulopathies, renal dysfunction, and the metabolic syndrome, (with an exception on age and sex) which may hinder the reliability and validity of the study outcomes. Excluded individuals also incorporated those with cancer, cardiovascular dysfunction, as well as those taking other medications which may influence inflammation or compromise the vascular endothelium [16,28]. This extends to individuals who were pregnant, lactating, or females who have reached menopause, and people with other disease/conditions implicated in oxidative stress, inflammation, and endothelial dysfunction.

### Ethical clearance

The study was approved by the Turfloop Research and Ethics Committee (TREC) under the University of Limpopo, South Africa, project number TREC/120/2023: IR (S1 File). Extending from a study by Hanser et al., (2021) [29] which obtained ethical approval from TREC under project number TREC/199/2016: PG (S2 File). Access to participant data for research purposes was obtained in June 2023 until October 2023. Further authorization to collect data from the Mankweng hospital and referral clinics was obtained from the Limpopo Department of Health and Social Development. Written consent was obtained from all the eligible study participants after having explained the study in both English and the local language (Sepedi) of the Mankweng community. Noteworthy, the written consent form was distributed to eligible participants in both the English and Sepedi language (S3 File). The consent form was approved by TREC under project number TREC/120/2023: IR.

### Data collection

A structured questionnaire successfully utilized by Hanser et al., (2022) [16] aligned with the aims and objectives of the present study was employed to collect the necessary sociodemographic and medical information about each participant. In addition, with the aid of the questionnaire we collected data on the lifestyle risk factors of the participants incorporating self-reported tobacco smoking and alcohol consumption. The participant’ medical file was evaluated for additional medical information incorporating duration on HAART.

### Blood collection

All blood samples were collected at a single point in time by a qualified nurse registered with the South African Nursing Council. Adherence for blood sampling and storage followed the protocol documented by Chen *et al*. (2019) [30]. Briefly, the blood sample was centrifuged to acquire serum and plasma samples. The blood samples were centrifuged at 3000rpm at a temperature of 15–24°C for 20 minutes to separate it into serum and plasma. Serum and plasma samples were then transferred into polypropylene microfuge tubes and kept in the bio-freezer at -85°C.

### Anthropometric data

Anthropometric measurements including height and weight used to calculate the Body Mass Index (BMI), waist circumference, together with systolic and diastolic blood pressure of each participant were measured by qualified and trained personnels. Adherence was given to the anthropometric measurement protocol by Andreacchi et al., (2021) [31]. Waist circumference was classified as a risk factor for CVDs at cut-off values of > 102cm for males and > 88cm for females [32]. Body mass index (BMI) was calculated as weight in kilograms (kg) divided by the square of the height in meters (m) (kg/m^2^). To evaluate the participants CVD-risk based on body weight, BMI cut-off points were classified as follows: <18.5 kg/m^2^ underweight, 18.5–24.9 kg/m^2^ normal weight, 25.0–29.9 kg/m^2^ overweight, 30.0–34.9 kg/m^2^ class I obesity, 35.0–39.9 kg/m^2^ class II obesity, ≥ 40 kg/m^2^ class III obesity [33]. Systolic blood pressure (SBP) and diastolic blood pressure (DBP) were measured using a digital automated Omron M3 BP monitor (OMRON Healthcare, Japan) Adhering to the manufacturer’s instructions. Noteworthy, blood pressure (BP) was measured twice on the right arm at a 5-minute interval when the participant was in a relaxed, upright seated position in a quiet room. Upon complete quantification, the overall BP reading was the average of the two BP measurements [16]. hypertension was classified by a SBP ≥ 140mmHg and/or a DBP ≥ 90mmHg [34].

### Analysis for HIV status and CD4^+^ count

All blood samples collected at the clinic were analysed for the presence of the HIV virus using an Alere Determine^™^ HIV-1/2 Ag/Ab combo kit (Abbott Medical Co Ltd., Japan), this was done according to manufacturer’s instructions. Cluster differentiation 4 positive (CD4^+^) count was determined on a factory calibrated Alere PIMA^™^ analyzer (Abbott Medical Co Ltd., Japan), according to the manufacturer’s instructions. Cluster differentiation 4 positive (CD4^+^) count was classified (as follows: normal ≥ 500 cells/mm^3^, diminished < 499 to 300 cells/mm^3^, AIDs <200 cells/mm^3^) in accordance with the World Health Organization guidelines on assessing immune status after or at HIV diagnosis [35].

### Cardiometabolic markers

The Cobas^®^ Integra 400 plus auto-analyzer (Systemic Liquicolour Reagent test kit, Germany) was used to quantify serum glucose and lipids including LDL-C, high-density lipoprotein cholesterol (HDL-C), triglycerides, and total cholesterol by means of enzymatic colorimetric methods. The manufacturer’s instructions, as provided in the laboratory training manual, were strictly adhered to (Systemic Liquicolour Reagent test kit, Germany). Systolic and diastolic blood pressure were measured to assess the participant’s CVD-risk, adherence was given to a similar protocol used by Woldu et al., (2022) [36]. Lipodystrophy was classified as elevated triglycerides ≥ 8.3mmol/L, diminished HDL-C < 2.2mmol/l for males and < 2.8mmol/L for females, total cholesterol > 5.2 mmol/L and elevated LDL-C > 4.91 mmol/L [32].

#### Fasting blood glucose

Using the enzymatic colorimetric approach involving hexokinase, glucose was quantified on the Cobas^®^ Integra 400 plus auto-analyzer (Roche Diagnostics). Briefly, these auto-quantifying technique involves the reaction between adenosine triphosphate (ATP) and a glucose molecule catalysed by hexokinase in the presence of a magnesium cofactor ion. The metabolites of this reaction are glucose-6-phoaphate (G6P) and adenosine diphosphate. Upon introducing nicotinamide adenine dinucleotide phosphate and glucose-6-phosphate dehydrogenase (G6PD) to the rection, G6PD oxidized G6P to yield 6-phosphogluconate and nicotinamide adenine dinucleotide phosphate (NADPH). The concentration of glucose is directly proportional to the concentration of NADPH generated by this reaction. Thus, we indirectly quantified the participants FBG by reading the concentration of NADPH at an absorbance of 340 nm. All reference values for the participants’ FBG were classified in accordance with the World Health Organization (WHO). Hypoglycaemia was reported at glucose levels < 3.9 mmol/L, with glucose levels between 3.9–5.6 mmol/L, 5.7–6.9 mmol/L, and ≥ 7.0 mmol/L being considered as normal, prediabetic, and diabetic, respectively [37].

#### Triglycerides

Triglycerides were quantified in sera using an enzymatic colorimetric technique (GPO/PAP) of a Systemic Liquicolor Reagent test kit (Germany), on the Cobas^®^ Integra 400 plus auto-analyzer. This assay’s technique is based on a modified Trinder colour reaction, which yields a linear endpoint reaction. Lipases were used to hydrolysed triglyceride to produce glycerol and free fatty acids. Glycerol was converted into glycerol-3-phosphate using glycerol kinases. Which was subsequently oxidized to dihydroxyacetone phosphate and hydrogen peroxide (H_2_O_2_) by glycerophosphate oxidase. Using peroxidase as a catalyst, H_2_O_2_ combined with 4-aminoantipyrine and 3,5-dichloro-2-hydroxybenzene to produce a red quinoneimine fluorescent colour. The concentration of triglycerides in solution were directly proportional fluorescent intensity of the generated colour at a wavelength of 510 nm [29].

#### High density lipoprotein cholesterol

High density lipoprotein cholesterol (HDL-C) was also quantified on the Cobas^®^ Integra 400 plus auto-analyser. Using an enzymatic technique involving cholesterol esterase and cholesterol oxidase linked with a polyethylene glycol on their amino groups (Systemic Liquicolor Reagent test kit, Germany). Cholesterol esters were quantitatively degraded using cholesterol esterase into free cholesterol and fatty acids. The free cholesterols were then oxidized by cholesterol oxidase to 4-cholestenone and H_2_O_2_ in the presence of oxygen molecules. In addition, H_2_O_2_ produced a fluorescent colour in which its intensity was directly proportional to the concentrations of HDL-C at a wavelength spectrum of 500nm [29].

#### Total cholesterol

Similar to quantifying triglycerides and HDL-C, the Cobas^®^ Integra 400 plus auto-analyser (Systemic Liquicolor Reagent test kit, Germany) was used to quantify total cholesterol using an enzymatic colorimetric method (CHOD/PAP). However, this technique was based on quantifying 4-cholestenone after enzymatic degradation of cholesterol esters by a cholesterol esterase. This was followed by the modification cholesterol using cholesterol oxidase and yielding H_2_O_2_ in a Trinder reaction. The fluorescent intensity of H_2_O_2_ were directly proportional to the concentration of cholesterol measured at a wavelength of 500nm [29].

#### Low density lipoprotein cholesterol

Serum levels of LDL-C were computerised automatically on the laboratory information system. The calculation used to determine LDL-C was structured as follow:

$$\text{LDL-C}=\text{Total}\;\text{cholesterol}-\left(\frac{\text{triglyceride}}{2.2}\right)+\text{HDL-C}$$

### Inflammatory and endothelial activation markers

Luminex bead-based multiplex immunoassay (EMD Millipore Corporation, Billerica, USA) was used to quantify inflammatory and endothelial activation biomarkers that included sP-selectin, sICAM-1, and sVCAM-1, following a similar protocol used by Eckels et al., (2013) [38] and Hanser, (2021) [29]. To date, there are no standard cut-off points for sP-selectin, sVCAM-1, and sICAM-1 both in HIV-negative persons and PLWH. Thus, in the present study we classify both inflammatory and endothelial markers as elevated using the cut-off points defined by the manufacturers protocol and existing literature by Hanser et al., (2022) [16]: sP-selectin > 50ng/mL, sICAM-1 ≥ 40ng/mL, and sVCAM-1 > 25ng/mL.

#### Analytical procedure for Luminex assays

A Luminex 200TM device equipped with a commercial bead-based multiplex kit was used to quantify all inflammatory and endothelial activation markers incorporating sP-selectin, sICAM-1, and sVCAM-1. Briefly, Luminex and flow cytometry share a similar standard protocol, although Luminex has an added program to sorts pre-coated, identical-sized analyte-specific beads that have been ligated with various mean fluorescence intensities (MFI) with varying amounts of red dye. To inspect the pre-coated, analyte-specific beads, Luminex 200 contains two lasers. The lasers look at the beads’ spectral properties (MFI, red dye content concentration), as well as the response involving the analyte (antibody) of the specific bead. Importantly, the Luminex device requires only a single 5μl blood sample which can contain a multitude of protein and cytokine targets, rendering the multiplex system capable of quantifying over 100 analytes at once. However, this is only possible if analytes of interest share similar dilution factors and other properties is to customize a bead-based multiplex kit to quantify different analytes at once.

The present study made use of the human CVD magnetic bead-based multiplex panel 2 (EMD Millipore Corporation, Billerica, USA, 2017) to quantify sP-selectin, sICAM-1, and sVCAM-1 simultaneously in 25μl serum samples. The analytes’ assay sensitivity falls within the pg/mL range, while the intra-assay and inter-assay coefficients of variation were less than 10% and 20%, respectively. To summarize, several sets of 150μl fluorescently dyed beads, one for each biomarker of interest, were combined with capture monoclonal antibodies unique to each indicator. In this study, for the human CVD magnetic bead-based panel 2 (HCVD2MAG-67K-03) we used serum sample serial dilution of 1:100. The entire 96-well plate was filled with 25μl of assay buffer. A preset well map was used to decide which amount of diluted serum, premixed beads solution, and monoclonal antibodies (25μl) to put on the EMD Millipore MILLIPLEX^®^ 96-well plate along with the 25μl quality controls and standards. The plate shaker was used to incubate the 96-well micro-plates for 12 hours at a temperature of 2 to 8°C at 600 rpm. The next day, the 96-well plate was incubated for 90 seconds in a magnetic plate before being washed three times in a 200μl wash buffer.

Thereafter, the 96-well plate was filled with fluorescent detection antibody combination, and it was incubated for one hour at 22°C and 600 rpm in a plate shaker. After adding 50μl of streptavidin-PE conjugate to the 96-well plate, it was incubated for 30 minutes. The Luminex 200MT apparatus was used to analyse the 96 well plates after they had been re-suspended in Luminex sheath fluid. To make sure the system was operating properly and ensuring data accuracy, tests for calibration and performance verification were conducted prior to the samples being ran. For data acquisition, the xPONENT^®^ software package 5.1 was employed. The study employed the Milliplex company’s xPONENT^®^ software program to obtain readings for each of the biomarkers. The software helps retrieve data on the analytes based on the milliplex beads.

### Statistical analysis

The International Business Machines Statistical Package for the Social Sciences (IBM SPSS) software (version 29.0) was used for data analysis. The normality of the data was determined using Pearson test of normality and quantile-quantile (Q-Q) plots. We further performed frequency distribution and descriptive statistics for categorical variables. Skewed variables including sP-selectin, sICAM-1, and sVCAM-1 were log-transformed which allowed the use of parametric statistical tests. Analysis of variance (ANOVA) were performed to compare means and standard deviations of the participant’s sociodemographic and clinical parameters. The relationship between inflammatory and endothelial activation markers was determined using Pearson’s and Partial correlations. Further associations between inflammatory and endothelial activation markers were determined using hierarchical multiple linear regression, while adjusting for age, BMI, and lipid parameters. A probability (p)-value of ≤ 0.05 was considered for significance for all statistical tests.

## Results

### Sociodemographic and clinical characteristics of participants

Majority of the participants enrolled in this study were females (65.9%) with a mean age (± SD) of 39.82 ± 13.04 (Table 1). The overall study population was classified into three groups, namely PLWH on HAART (n = 55), HAART-naïve (n = 29), and the HIV-negative controls (n = 48). Human immunodeficiency virus related participants characteristics including CD4+ count and HAART drug regimen combinations are described on S4 File. Notably, serum levels of LDL-C were significantly higher in PLWH on HAART and HIV-negative persons (p = 0.007) as compared to HAART-naïve PLWH. Similarly, total cholesterol levels were significantly higher in PLWH on HAART (p<0.001) and HIV-negative persons (p = 0.003) when compared to HAART-naïve PLWH. Serum levels of HDL-C in PLWH on HAART were comparable to those of the HIV-negative control but were significantly higher (p = 0.002) than those observed in the HAART-naïve group (Table 1). There was no significant difference in mean systolic (p = 0.389) and diastolic (p = 0.387) blood pressure, and triglycerides across the study groups. As expected, the mean (± SD) CD4^+^ count was higher in PLWH on HAART (434.20 ± 209.69) compared to the HAART-naïve group (331.19 ± 264.23). Majority of the PLWH on HAART were on 1^st^ line regimen consisting of TDF/FTC/EFV (83.6%), while 2^nd^ line regimen consisted only of 3TC/AZT/LPV-r (16.4%) (S4 File). Most of the PLWH on HAART, 34 (64.2%) had been taking the treatment for ≥3 years (S4 File).

**Table 1:** Socio-demographic and clinical parameters for the different study participants.

Total population (N = 132)					
Variables		HAART-naïve (n = 29)	PLWH on HAART (n = 55)	HIV-negative control (n = 48)	p-value
**Pearson Chi-square**					
Gender (n; %)	Male	14 (48.3)	18 (32.70	13 (27.1)	0.158
	Female	15 (51.7)	37 (67.3)	35 (72.9)	
Smoking (n; %)	Yes	8 (27.6)	11 (20.0)	7 (14.6)	0.379
	No	21 (72.4)	44 (80.0)	41 (85.4)	
**One-way ANOVA**					
Age		39.59 ± 11.83	42.87 ± 10.68	36.46 ± 15.38	**0.006** *
BMI (Kg/m^2^)		23.20 ± 3.56	25.59 ± 6.10	27.72 ± 5.50	**<0.001** *
SBP (mmHg)		118.79 ± 14.76	122.64 ± 21.39	118.15 ± 13.80	0.389
DBP (mmHg)		73.41 ± 9.30	76.04 ± 9.78	74.06 ± 8.71	0.387
HDL-C (mmol/L)		1.21 ± 0.49	1.47 ± 0.35	1.33 ± 0.44	**0.002** *
LDL-C (mmol/L)		1.85 ± 0.81	2.46 ± 0.86	2.50 ± 1.00	**0.004** *
Total cholesterol (mmol/L)		3.63 ± 0.74	4.45 ± 0.94	4.39 ± 1.15	**<0.001** **
Triglycerides (mmol/L)		1.27 ± 0.75	1.13 ± 0.42	1.21 ± 0.63	0.570
CD4* count		331.19 ± 264.23	434.20 ± 209.69	-	0.066

* Significant at p<0.01;

** Significant at p<0.001.

**Post-Hoc test:** People living with HIV (PLWH) on HAART were older (p = 0.004) than HIV-negative persons as compared to HAART-naïve PLWH (p = 0.371); BMI was higher (p<0.001) in HIV-negative persons compared to the HAART-naïve PLWH and PLWH on HAART (p = 0.200); HDL-C was higher (p = 0.002) in PLWH on HAART compared to the HAART-naïve PLWH, but comparable with those observed in HIV-negative persons (p = 0.116); LDL-C was higher in PLWH on HAART (p = 0.009) and HIV-negative persons (p = 0.007) compared to the HAART naïve PLWH; total cholesterol was higher in PLWH on HAART (p<0.001) and HIV-negative persons (p = 0.003) compared to HAART naïve PLWH. BMI—Body Mass Index; SBP—systolic blood pressure; DBP—diastolic blood pressure; HDL-C—high density lipoprotein cholesterol; LDL-C—low density lipoprotein cholesterol; CD4^+^–cluster differentiation 4 positive.

### Differences in serum levels of inflammatory and endothelial markers across the study groups

The present study evaluated levels of sP-selectin as one of the prominent markers of inflammation, together with sICAM-1 and sVCAM-1 as potential markers of endothelial activation across the study groups. The results showed that mean (± SD) sP-selectin 0.51 ± 0.48 was higher (p = 0.017) in HAART-naïve PLWH compared to PLWH on HAART with a mean (± SD) value of 0.24 ± 0.10 (Table 2). Similarly, mean (± SD) sP-selectin was higher in HAART-naïve PLWH compared to the HIV-negative persons with a mean (± SD) of 0.29 ± 0.24. There were no significant differences in serum levels of sVCAM-1 across the study groups (Table 2). People living with HIV who were HAART-naïve displayed a higher level of sICAM-1 (p = 0.004) with a mean (± SD) of 0.01 ± 0.33, compared to HIV-negative persons with a mean (± SD) of -0.39 ± 0.72 (Table 2).

**Table 2:** Differences in Inflammatory and endothelial markers across the study population groups.

Total population (N = 132)				
Variables	HAART-naïve (n = 29)	PLWH on HAART (n = 55)	HIV-negative control (n = 48)	p-value
**One-way ANOVA**				
sP-selectin (Mean ± SD)	0.51 ± 0.48	0.24 ± 0.10	0.29 ± 0.24	**0.011** *
sVCAM-1 (Mean ± SD)	0.61 ± 0.49	0.76 ± 0.45	0.77 ± 0.73	0.371
sICAM-1 (Mean ± SD)	0.01 ± 0.33	-0.12 ± 0.71	-0.39 ± 0.72	**0.006** **

* Significance at p<0.05;

** Significant at p<0.01.

**Post-Hoc test:** Soluble P-selectin was significantly higher (p = 0.017) in HAART-naïve PLWH compared to PLWH on HAART. Soluble sICAM-1 was significantly higher (p = 0.004) in HAART-naïve PLWH compared to the HIV-negative persons. Both Welch and Brown-Forsythe tests were not significant for sVCAM-1.

### Inflammatory status, and potential correlation to endothelial activation in PLWH on HAART

The current study also evaluated the correlation between sP-selectin as an inflammatory mediator, and sVCAM-1 and sICAM-1 in PLWH on HAART. The results showed that sP-selectin was positively correlated to sVCAM-1 in PLWH on HAART (r = 0.469; p<0.001) (Table 3). There was no significant correlation between sP-selectin and sICAM-1 in PLWH on HAART (r = 0.242; p = 0.075). Duration on HAART was negatively correlated to sICAM-1 (r = -0.204; p = 0.041) (Table 3).

**Table 3:** Correlation between inflammatory status, and endothelial activation function amongst different study groups.

Total population (N = 132)																	
Variables		Crude								Adjusted							
		Total population (n = 132)		HAART-naïve (n = 29)		HAART-exposed (n = 55)		HIV-negative control (n = 48)		Total population (n = 132)		HAART-naïve (n = 29)		HAART on HAART (n = 55)		HIV-negative control (n = 48)	
		sVCAM-1	sICAM-1	sVCAM-1	sICAM-1	sVCAM-1	sICAM-1	sVCAM-1	sICAM-1	sVCAM-1	sICAM-1	sVCAM-1	sICAM-1	sVCAM-1	sICAM-1	sVCAM-1	sICAM-1
sP-selectin	r	0.014	0.198*	-0.647***	0.155	0.469***	0.213	0.550***	0.456**	0.056	0.196	-0.678***	0.198	0.467**	0.217	0.697***	0.475**
	p	0.877	**0.014**	**<0.001**	0.422	**<0.001**	0.108	**<0.001**	**0.002**	0.531	**0.027**	**<0.001**	0.354	**<0.001**	0.130	**<0.001**	**0.001**
HAART duration	r	-	-	-	-	-0.204	-0.277*	-	-	-	-	-	-	-0.155	-0.337*	-	-
	p	-	-	-	-	0.135	**0.041**	-	-	-	-	-	-	0.281	**0.017**	-	-

* Significant at p<0.05.;

** Significant at p = 0.001;

*** Significant at p<0.001.

Adjusted confounders: age; Body Mass Index (BMI); total cholesterol; high density lipoprotein cholesterol (HDL-C); and low-density lipoprotein cholesterol (LDL-C).

After adjusting for confounders incorporating age, total cholesterol, LDL-C, BMI, and HDL-C, sP-selectin was positively correlated with sVCAM-1 in PLWH on HAART (r = 0.467; p<0.001) (Table 3). Duration on HAART was negatively correlated with sICAM-1 (-0.337; p = 0.017) (Table 3). Hierarchical multiple linear regression indicated that sP-selecting was independently associated with the expression of sVCAM-1 (β = 0.445; p<0.001), even after adjusting for confounders (β = 0.475; p = 0.001) in PLWH on HAART (Table 4). Duration on HAART had no significant association with endothelial markers sVCAM-1 (β = -0.105; p = 0.405), sICAM-1 (β = -0.235; p = 0.086), however, we observed a significant association (p = 0.035) after adjusting for confounders (Table 4).

**Table 4:** Hierarchical multiple linear regression on factors affecting markers of endothelial activation in people living with the human immunodeficiency virus (PLWH) on highly active antiretroviral therapy (HAART).

PLWH on HAART (n = 55)								
Variables	Crude				Adjusted			
	sVCAM-1 (Adj R^2^ = 0.201; p = 0.001)		sICAM-1 (Adj R^2^ = 0,077; p = 0.47)		sVCAM-1 (Adj R^2^ = 0.202; p = 0.003)		sICAM-1 (Adj R^2^ = 0.128; p = 0.034)	
	β(95%CI)	p	β(95%CI)	p	β(95%CI)	p	β(95%CI)	p
sP-selectin	0.445 (0.324; 1.156)	**<0.001** ***	0.190 (-0.210; 1.213)	0.163	0.475 (0.327; 1.251)	**0.001** **	0.157 (-0.375; 1.203)	0.297
HAART duration	-0.105 (-0.04; 0.002)	0.405	-0.235 (-0.008; 0.01)	0.086	-0.052 (-0.003; 0.002)	0.705	-0.321 (-0.010; 0.000)	**0.035**

*** Significant at p<0.001;

** significant at p = 0.001;

* significant at p<0.05.

Adjusted confounders: age; Body Mass Index (BMI); total cholesterol; high density lipoprotein cholesterol (HDL-C); and low-density lipoprotein cholesterol (LDL-C).

### Inflammatory status, and potential correlation to endothelial activation in HAART-naïve participants

The levels of sP-selectin, in correlation with sVCAM-1 and sICAM-1 was also evaluated in HAART-naïve PLWH. The results showed that sP-selectin was negatively correlated (r = -0.647; p<0.001) to sVCAM-1 in the HAART-naïve PLWH (Table 3). There was no significant correlation between sP-selectin and sICAM-1 in HAART-naïve PLWH (r = 0.155; p = 0.422). After adjusting for confounders incorporating age, total cholesterol, LDL-C, and BMI, and HDL-C, sP-selectin remained negatively correlated with sVCAM-1 (r = -0.678; p<0.001) in HAART-naïve PLWH (Table 3).

### Inflammatory status, and potential correlation to endothelial activation in participants without HIV

The levels of sP-selectin in comparison to sVCAM-1 and sICAM-1 were also evaluated in HIV-negative persons. The results showed that sP-selectin was positively correlated to sVCAM-1 in participants without HIV (r = 0.550, p<0.001) (Table 3). Similarly, sP-selectin was positively correlated with sICAM-1 (r = 0.456; p = 0.002) in the HIV-negative persons (Table 3). After adjusting for confounders incorporating age, total cholesterol, LDL-C, and BMI, and HDL-C, sP-selectin was positively correlated with sVCAM-1 in HIV-negative persons (r = 0.6697; p<0.001) (Table 3). Similarly, after adjusting for confounders, sP-selectin remained positively correlated with sICAM-1 (r = 0.475; p = 0.001) in HIV-negative persons.

### Inflammatory status, and potential correlation to endothelial activation in the total study population

It remained important to evaluate serum levels of sP-selectin as an inflammatory mediator in comparison with the markers of endothelial activation sICAM-1 and sVCAM-1 across the total study population. Soluble P-selectin was correlated to sICAM-1 (r = 0.198; p = 0.014). Similarly, after adjusting for confounding factor sP-selectin was correlated to sICAM-1 (r = 196; p = 0.027). There was no significant correlation between sP-selectin and sVCAM-1 (r = 0.014; p = 0.877), even after adjusting for confounding factors (r = 0.056; p = 0.531).

## Discussion

The use of HAART remains effective at controlling HIV replication and preventing the progression of this virus to acquired immunodeficiency syndrome (AIDS), albeit elevated levels of inflammation remain persistent in PLWH [39]. The reasons for persistent inflammation in some PLWH despite successful treatment are complex and not fully understood [40–42]. Thus, there is a growing need to understand the pathological consequences of inflammation in PLWH, especially describing the potential contribution to the development of CVDs; perhaps highlighting the significance of the current study in reporting on the potential role of sP-selectin as an inflammatory mediator contributing to the initiation of endothelial activation in PLWH on HAART. Additionally, the study explores the impact of the duration of HAART exposure on markers of endothelial activation.

Soluble P-selectin has been shown to mediate the interaction between activated thrombocytes and other immune cells with endotheliocytes, which contributes to endothelial activation marked by an enhanced expression of sICAM-1 and sVCAM-1 [43,44]. That is, during early inflammation activated platelets express sP-selectin which ligates to circulating leukocytes incorporating monocytes and neutrophils [45,46]. This phenomenon causes leukocytes to circulate closer to the vascular endothelium predisposing to their adhesion on activated endothelial cell expressed P-selectin. Consequently, rendering the vascular endothelium permeable to circulating monocytes, neutrophils, and polyunsaturated lipid products which induce the formation of inflammatory foam cells and atherosclerotic plaques [47]. Moreover, evidence on animal models exist indicating that sP-selectin inhibition may promote endothelial recovery potentially preventing the development of CVDs [45,46]. Such evidence signifies the significant role of sP-selectin in mediating inflammation induced endothelial activation, especially in people living with adverse inflammatory conditions such as HIV.

**Fig 1:**
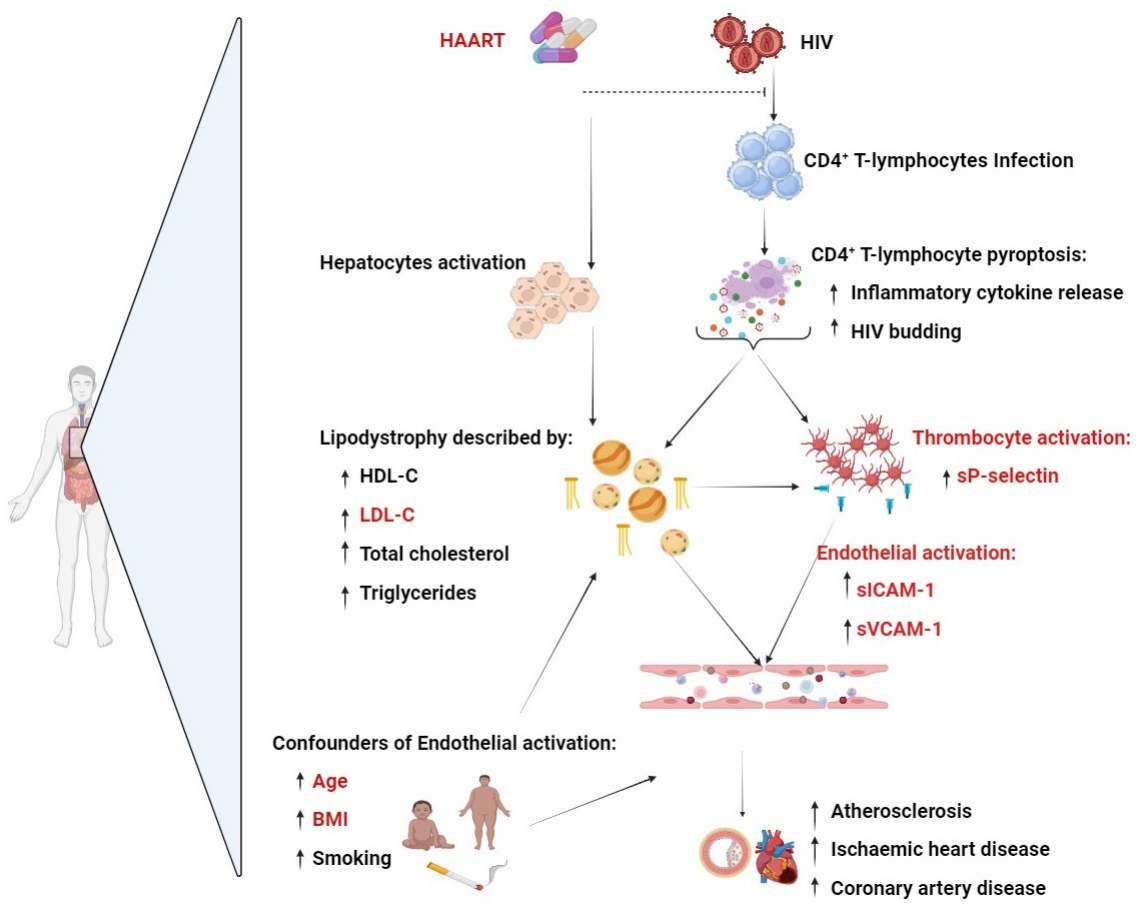
Human immunodeficiency virus (HIV) mediated cardiovascular diseases (CVDs). The human immunodeficiency virus activates thrombocytes which potentially release sP-selectin, an inflammatory mediator that triggers endothelial activation preceding CVDs. Concurrently, the use of highly active antiretroviral therapy (HAART) for viral suppression may potentially dysregulate cholesterol metabolism, causing increased levels of vLDL-C escalating to an elevated CVD-risk. Such pathological changes are associated with enhanced levels of endothelial activation markers such as sICAM-1 and sVCAM-1, a precursor event of CVDs. Furthermore, age, high Body Mass Index (BMI), and frequent smoking remain major contributing risk factors in endothelial activation and an elevated risk of developing CVDs, including atherosclerosis.

In the present study, PLWH on HAART showed diminished serum levels of sP-selectin contrary to their HAART-naïve and HIV-negative contour parts. Noteworthy, PLWH on HAART presented with elevated levels of sVCAM-1 compared to HAART-naïve PLWH. These findings on elevated sVCAM-1 give evidence of endothelial activation in PLWH on HAART. Furthermore, even after adjusting for confounding factors incorporating LDL-C, age, total cholesterol, and BMI, an increase in sP-selectin was consistent with elevated levels of sVCAM-1 among PLWH on HAART. Suggesting that sP-selectin could potentially function as an inflammatory mediator, driving endothelial activation in PLWH on HAART. It is also worth noting that our findings indicate that HAART-naïve PLWH exhibit elevated levels of sP-selectin compared to those on HAART. This may suggest that the HIV infection may play a role in thrombocyte activation, leading to an elevated release of sP-selectin. Nonetheless, our results support the notion that elevated level of sP-selectin may predict CVD-risk (Fig 1), especially platelet activation in PLWH [23,48]. Further motivating the need to understand the link between elevated inflammation and broader indicators of CVD-risk such as measurement of blood lipid profiles.

Our findings further showed that PLWH on HAART displayed increased levels of LDL-C and total cholesterol, which are known contributors to the accumulation of atherosclerotic plaques to elevate CVD-risk in people with diverse medical conditions [49,50]. Elevated LDL-C levels may also trigger endothelial activation by stimulating thrombocytes to release sP-selectin in individuals at increased CVD-risk [51]. Consistent with our findings, Darwin et al., (2020) [52] reported that dysregulated lipid profiles may amplify the expression of sP-selectin and promote leukocyte adhesion in patients with CVD. The abnormal expression of sP-selectin resulting from the combined effects of abnormal lipid profile accumulation and HIV infection may intensify the strain on endothelial activation, potentially exacerbating complications in CVD (Fig 1). Hence, our findings highlight the prognostic value of sP-selectin to detect endothelial activation in PLWH on HAART [53,54]. This is especially important to consider since our research did not identify any correlation between levels of sP-selectin and makers of endothelial activation in HAART-naïve PLWH. This is in line with previous research already indicating that the duration on HAART might exacerbate endothelial activation, characterized by an elevated expression of sICAM-1 and sVCAM-1 [55].

Overall, our results indicate that PLWH are at an elevated risk of CVDs compared to individuals without this condition [56]. The use of HAART appears to have protective effects against inflammation and sustained endothelial activation [57]. However, in the long run, HAART may contribute to chronic inflammation consistent with dysregulation of lipid profiles, predisposing the vascular system to sustained endothelial activation [58]. From our findings, sP-selectin remains a critical mediator between inflammation and endothelial activation in PLWH. It is important to also note that inflammation may be exacerbated by confounding factors incorporating age and increased BMI in PLWH on HAART [59,60] (Fig 1). From our study population, PLWH on HAART were older with a higher BMI than both HAART-naïve PLWH and HIV-negative persons. In addition to these confounders, the incidence of hypertension, one of the major risk factors of CVDs has been reported to be high in PLWH [61]. Contrary to this report, the was no significant differences in mean systolic and diastolic blood pressure across our study groups. Within the sub-Saharan African rural population, there is very limited information on the regulation of sP-selectin, together with sICAM-1 and sVCAM-1, or any other CVD-related makers in PLWH. However, some evidence has emerged from other developing countries such as Brazil, where they showed that PLWH on HAART presented a higher expression of sP-selectin as opposed to HIV-negative persons [62]. Further highlighting a need to outline the role of sP-selectin during the pathogenesis of inflammation or related development of endothelial dysfunction in PLWH on HAART.

## Study strengths and limitations

The current study reports on the potential connection between sP-selectin as an inflammatory mediator and endothelial markers incorporating sICAM-1 and sVCAM-1 in people living with HIV (PLWH). Our findings delineated both the potential advantages and drawbacks of administering HAART on the vascular endothelium and the associated risks of CVD. However, the study has several noteworthy limitations. Incorporating an unequal sample size among our study groups due to limited resources and challenges in recruiting participants for HIV-related research in our community of choice. The recruitment of participants to partake in the study regardless of their HART regimen combination, leading to the data on drugs not being pooled purposefully, thus limiting us from grouping participants based on their treatment regimen and running statistical tests to compare their association with our biomarkers of interest. Moreover, we did not investigate lifestyle factors due to these being beyond the scope of the current research. However, we acknowledge that beside pharmacodynamics, these factors, especially unhealthy diet, physical inactivity, and harmful substance use may impact/mitigate vascular health processes, thereby inducing inflammation and oxidative stress implicated in impairing endothelial function [63,64]. Future research should also consider these afore-mentioned factors and further incorporate more markers of inflammation and endothelial function to comprehensively address the physiological pathways linking these two conditions, within a broader study population.

## Conclusion

Soluble P-selectin, as an inflammatory mediator, may promote sustained endothelial activation through enhancing the expression of sVCAM-1 before endothelial dysfunction in PLWH on HAART. These outcomes might be intensified by HAART induced elevated serum levels of LDL-C in PLWH [65]. We are also not ruling out that other confounding factors, incorporating age, obesity, and lipid dysregulation may interfere with the efficacy of HAART, leading to increased CVD-risk in PLWH. There remains a need for future studies to validate our findings especially whether specific HAART drug combinations may have detrimental effects on leukocytes and endotheliocytes preceding CVDs in PLWH.

## Supporting information

S1 File Turfloop Research and Ethics Committee (TREC) approval letter, project no. TREC/120/2023: IR. (PDF)

S2 File TREC approval letter, project no. TREC/199/2016: PG. (PDF)

S3 File TREC approved consent form. (DOCX)

S4 File Human immunodeficiency virus and highly active antiretroviral therapy (HAART) related clinical parameters of participants. (DOCX)

S5 File Raw manuscript data. (XLSX)
