# Supplementary material for: Soluble P-selectin as an inflammatory mediator potentially influencing endothelial activation in people living with HIV in sub-rural areas of Limpopo, South Africa
Source: PLoS One. 2024 Nov 27;19(11):e0310056. doi: 10.1371/journal.pone.0310056 (PMC11602056; doi:10.1371/journal.pone.0310056)
Supplement: S1 File — (PDF) [file pone.0310056.s001.pdf]

University of Limpopo
Department of Research Administration and Development
Private Bag X1106, Sovenga, 0727, South Africa
Tel: (015) 268 4713, Fax: (015) 268 2306, Email: moore.hutamo@ul.ac.za

TURFLOOP RESEARCH ETHICS COMMITTEE

ETHICS CLEARANCE CERTIFICATE

MEETING: **22 May 2023**

PROJECT NUMBER: **TREC/120/2023: IR**

PROJECT:

**Title:**	Assessing the detrimental effects of oxidative stress and inflammation responsible for endothelial dysfunction in HIV positive patients.
**Researcher:**	H Mokoena
**Supervisor:**	Dr S Hanser
**Co-Supervisor/s:**	Prof PV Dludla (SAMRC)
**School:**	Molecular and Life Sciences
**Degree:**	Master of Science in Physiology

PROF D MAPOSA
CHAIRPERSON: TURFLOOP RESEARCH ETHICS COMMITTEE

The Turfloop Research Ethics Committee (TREC) is registered with the National Health Research Ethics Council, Registration Number: **REC-0310111-031**

Note:

i) **This Ethics Clearance Certificate will be valid for one (1) year, as from the abovementioned date. Application for annual renewal (or annual review) need to be received by TREC one month before lapse of this period.**

ii) **Should any departure be contemplated from the research procedure as approved, the researcher(s) must re-submit the protocol to the committee, together with the Application for Amendment form.**

iii) **PLEASE QUOTE THE PROTOCOL NUMBER IN ALL ENQUIRIES.**
