# Supplementary material for: Soluble P-selectin as an inflammatory mediator potentially influencing endothelial activation in people living with HIV in sub-rural areas of Limpopo, South Africa
Source: PLoS One. 2024 Nov 27;19(11):e0310056. doi: 10.1371/journal.pone.0310056 (PMC11602056; doi:10.1371/journal.pone.0310056)
Supplement: S2 File — (PDF) [file pone.0310056.s002.pdf]

University of Limpopo
Department of Research Administration and Development
Private Bag X1106, Sovenga, 0727, South Africa
Tel: (015) 268 2212, Fax: (015) 268 2306, Email:noko.monene@ul.ac.za

TURFLOOP RESEARCH ETHICS COMMITTEE CLEARANCE CERTIFICATE

MEETING: **05 July 2016**

PROJECT NUMBER: **TREC/119/2016: PG**

<u>PROJECT:</u>

Title: Investigating the effects of Haart on early markers of cardiovascular disease among HIV-Positive patients in the Mankweng District, Limpopo Province

Researcher: Mr S Hanser
Supervisor: Dr M Van Staden
Co-Supervisor: Dr ELJ Erasmus
Prof MM Moraba
School: Molecular and Life Sciences
Degree: PhD in Physiology and Environmental Health

PROF TAB MASHEGO
CHAIRPERSON: TURFLOOP RESEARCH ETHICS COMMITTEE

The Turfloop Research Ethics Committee (TREC) is registered with the National Health Research Ethics Council, Registration Number: **REC-0310111-031**

Note:

i) **Should any departure be contemplated from the research procedure as approved, the researcher(s) must re-submit the protocol to the committee.**

ii) **The budget for the research will be considered separately from the protocol.**
PLEASE QUOTE THE PROTOCOL NUMBER IN ALL ENQUIRIES.
