# Supplementary material for: Soluble P-selectin as an inflammatory mediator potentially influencing endothelial activation in people living with HIV in sub-rural areas of Limpopo, South Africa
Source: PLoS One. 2024 Nov 27;19(11):e0310056. doi: 10.1371/journal.pone.0310056 (PMC11602056; doi:10.1371/journal.pone.0310056)
Supplement: S3 File — (DOCX) [file pone.0310056.s003.docx]

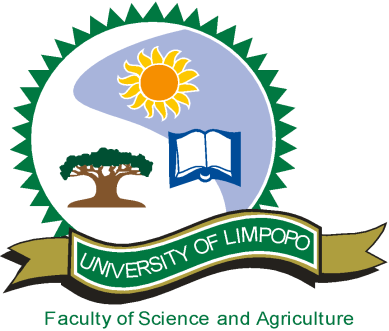


UNIVERSITY OF LIMPOPO, ETHICS COMMITTEE

**Study title:** Assessing the detrimental effects of oxidative stress and inflammation implicated in endothelial dysfunction in HAART-exposed and HAART-naïve HIV-positive patients.

**Project leaders:**

Dr. S. Hanser, Tel: 015 268 4189, E-mail: sidney.hanser@ul.ac.za

Prof. P.V. Dludla, Tel: 021 938 0333, E-mail: phowayinkosi.dludla@mrc.ac.za

Student: Mr. H. Mokoena, Tel: 015 268 4010, haskly.mokoena@ul.ac.za

**Subject:** Consent form

This study investigates the association between markers of oxidative stress and inflammation that are implicated in exacerbating endothelial dysfunction in HIV patients. Furthermore, the association between endothelial function and cardiovascular risk factors is explored. The study deals with the effect of HIV and highly active antiretroviral therapy on the human body. During this study, the research team will collect participant information incorporating lifestyle factors including but not limited to the use of alcohol and smoking, demographic factors such as educational and socio-economic status using a detailed questionnaire. Furthermore, the research team will request to take anthropometric measurements incorporating but not limited to height, weight, and waist circumference. Invasive measurements will include blood withdrawal using standard clinical syringes. All these measurements will be collected in health facilities around the Mankweng District, taking into consideration clinics and hospitals recommended to the participant by their medical practitioner. The procedure or treatment envisaged may hold some risk for me that cannot be foreseen at this stage.

The Ethics Committee has approved that individuals may be approached to participate in the study. The experimental protocol, i.e., the extent, aims and methods of the research, has been explained to me. The protocol sets out the risks that can be expected as well as possible discomfort for persons participating in the research, an explanation of the anticipated advantages for myself or others that are reasonably expected from the research and alternative procedures that may be to my advantage. I will be informed of any new information that may become available during the research that may influence my willingness to continue my participation. Access to the records that pertain to my participation in the study will be restricted to persons directly involved in the research. Any questions that I may have regarding the research, or related matters, will be answered by the researchers. If I have any questions about, or problems regarding the study, or experience any undesirable effects, I may contact a member of the research team.

Participation in this research is voluntary and I can withdraw my participation at any stage. If any medical problem is identified at any stage during the research, or when I am vetted for participation, such conditions will be discussed with me in confidence by a qualified person and/or I will be referred to my doctor. I indemnify the University of Limpopo and all persons involved with the above project from any liability that may arise from my participation in the above project or that may be related to it, for any reasons, including irresponsibility on the part of the mentioned persons. I am fully aware that the results of this project will be used for scientific purposes and may be published. I agree to this, provided my privacy is guaranteed.

I, ………………………. hereby voluntarily consent to participate in this project.

____________________________ ______________________________

Signature of the participant Date

______________________________ ______________________________

Signature of the witness Date

______________________________ ______________________________

Signature of researcher Date

______________________________

Place

WRITTEN CONSENT FORM TRANSLATED INTO SEPEDI, THE NATIVE LANGUAGE OF THE MANKWENG COMMUNITY IN LIMPOPO.

YUNIBESITHI YA LIMPOPO, KOMITI YA BOITSHWARO

Thaetlele: Go hlahloba ditlamorago tše kotsi tša kgateletšego ya oxidative le go ruruga tšeo di akaretšwago go se šome gabotse ga endothelial go balwetši bao ba pepentšhitšwego HAART le bao ba se nago HIV.

**Baetapele ba diprotšeke:**

Dr. S. Hanser, Tel: 015 268 4189, Mogala: sidney.hanser@ul.ac.za

Mop. P.V. Dludla, Tel: 021 938 0333, Mogala: phowayinkosi.dludla@mrc.ac.za.

Mna. H. Mokoena, Tel: 015 268 4010, Mogala: haskly.mokoena@ul.ac.za

**Hlogotaba:** Foromo ya tumelelo

Thuto ye e nyakišiša tswalano magareng ga maswao a kgateletšego ya oxidative le go ruruga ao a akaretšwago go gakatša go se šome gabotse ga endothelial go balwetši ba HIV. Go feta moo, go hlahlobja tswalano magareng ga modiro wa endothelial le mabaka a kotsi a pelo le methapo. Thuto e lebane le mafelelo a kalafo ya HIV le antiretroviral yeo e šomago kudu mmeleng wa motho. Nakong ya nyakišišo ye, sehlopha sa nyakišišo se tla kgoboketša tshedimošo ya batšwasehlabelo yeo e akaretšago mabaka a mokgwa wa bophelo go akaretšwa eupša e sego fela tšhomišo ya bjala le go kgoga, mabaka a palo ya batho go swana le maemo a thuto le a ekonomi ya leago ka go šomiša lenaneopotšišo leo le nago le dintlha ka botlalo. Go feta fao, sehlopha sa nyakišišo se tla kgopela go tšea dikelo tša anthropometric tšeo di akaretšago eupša e sego fela botelele, boima le sedikologo sa letheka. Ditekanyo tše di hlaselago di tla akaretša go ntšhwa ga madi ka go diriša disirinji tše di tlwaelegilego tša kalafo. Ditekanyo tše ka moka di tla kgoboketšwa mafelong a maphelo go dikologa Selete sa Mankweng, go elwa hloko dikliniki le dipetlele tšeo di šišinywago go motšwasehlabelo ke ngaka ya bona ya kalafo. Tshepedišo goba kalafo yeo e akantšwego e ka swara kotsi e itšego go nna yeo e ka se bonwego e sa le pele mo nakong ye. Komiti ya Boitshwaro e dumeletše gore batho ka o tee ka o tee ba ka batamelwa gore ba tšee karolo nyakišišong ye. Protocol ya teko, i.e., bogolo, maikemišetšo le mekgwa ya nyakišišo, e hlalošitšwe go nna. Prothokhole e hlagiša dikotsi tšeo di ka letelwago gammogo le go se iketle mo go kgonegago go batho bao ba tšeago karolo nyakišišong, tlhalošo ya mehola yeo e letetšwego go nna goba ba bangwe yeo e letetšwego ka mo go kwagalago go tšwa nyakišišong le ditshepedišo tše dingwe tšeo di ka bago le mohola wa ka. Ke tla tsebišwa ka tshedimošo efe goba efe ye mpsha yeo e ka bago gona nakong ya nyakišišo yeo e ka tutuetšago go ikemišetša ga ka go tšwetša pele go tšea karolo ga ka. Phihlelelo ya direkhoto tšeo di lebanego le go tšea karolo ga ka nyakišišong e tla lekanyeletšwa go batho bao ba amegago thwii nyakišišong. Dipotšišo dife goba dife tšeo nka bago le tšona mabapi le nyakišišo, goba ditaba tše di amanago le yona, di tla arabja ke banyakišiši. Ge ke na le dipotšišo mabapi le, goba mathata mabapi le nyakišišo, goba ke itemogela ditlamorago dife goba dife tše di sa rategego, nka ikgokaganya le leloko la sehlopha sa nyakišišo.

Go tšea karolo nyakišišong ye ke ga boithaopo gomme nka gogela morago go tšea karolo ga ka legatong lefe goba lefe. Ge bothata bofe goba bofe bja kalafo bo lemogwa mogatong ofe goba ofe nakong ya nyakišišo, goba ge ke hlahlobja gore ke tšee karolo, maemo a bjalo a tla ahlaahlwa le nna ka sephiri ke motho yo a nago le maswanedi le/goba ke tla romelwa ngakeng ya ka. Ke šireletša Yunibesithi ya Limpopo le batho ka moka bao ba amegago ka projeke ye e lego ka mo godimo go maikarabelo afe goba afe ao a ka tšwelelago ka lebaka la go tšea karolo ga ka mo protšekeng ye e lego ka mo godimo goba yeo e ka amanago le yona, ka mabaka afe goba afe, go akaretšwa go hloka maikarabelo ka lehlakoreng la batho bao go boletšwego ka bona. Ke tseba ka botlalo gore dipoelo tša projeke ye di tla šomišetšwa merero ya mahlale gomme di ka phatlalatšwa. Ke dumelelana le se, ge feela sephiri sa ka se kgonthišeditšwe.

NNA, ………………………. ka go dumela ka boithaopo go tšea karolo mo protšekeng ye.

____________________________ ______________________________

Mosaeno wa motšwasehlabelo Letšatšikgwedi

______________________________

______________________________

Mosaeno wa hlatse Letšatšikgwedi

______________________________ ______________________________

Mosaeno wa monyakišiši Letšatšikgwedi

______________________________

Lefelo
