# Supplementary material for: Soluble P-selectin as an inflammatory mediator potentially influencing endothelial activation in people living with HIV in sub-rural areas of Limpopo, South Africa
Source: PLoS One. 2024 Nov 27;19(11):e0310056. doi: 10.1371/journal.pone.0310056 (PMC11602056; doi:10.1371/journal.pone.0310056)
Supplement: S4 File — (DOCX) [file pone.0310056.s004.docx]

## **Supplementary file 1.**

Table 2: Human immunodeficiency virus and highly active antiretroviral therapy (HAART) related clinical parameters of participants.

|  | | Total population | HAART-naïve | HAART-exposed | *p*-value |
| --- | --- | --- | --- | --- | --- |
| CD4^+^ count | <200 | 7 (5.3) | - | 7 (12.1) | **0.054** |
|  | 201 – 500 | 15 (11.4) | - | 15 (25.9) |  |
|  | 501 – 1500 | 14 (10.6) | 1 (3.4) | 13 (22.4) |  |
|  | ≥1501 | 2 (1.5) | - | 2 (3.4) |  |
| 1^st^ line regimen | TDF/FTC/EFV | 42 (31.8) | - | 42 (72.4) | - |
|  | AZT/3TC/NVP | 2 (1.5) | - | 2 (3.4) |  |
|  | Not disclosed | 2 (1.5) | - | 2 (3.4) |  |
| 2^nd^ line regimen | 3TC/AZT/LPV-r | 9 (6.8) | - | 9 (15.5) | - |
| HAART duration | <3 years | 40 (30.3) | - | 40 (69.0) | **0.054** |
|  | ≥3 years | 13 (9.8) | - | 13 (22.4) |  |

* Significant at p≤0.05. CD4^+^ – cluster differentiation 4 positive; 3TC – Lamivudine; EFV – Efavirenz; FTC – Emtricitabine; AZT – Zidovudine; LPV-r – Lopinavir boosted ritonavir; NVP – Nevirapine; TDF – Tenofovir disoproxil fumarate.
